# Sulfadiazine crystalluria in a patient with lupus nephritis

**DOI:** 10.1515/almed-2022-0046

**Published:** 2022-06-16

**Authors:** Maria Jesús Ruiz Álvarez, Sergio Molina Blas, Marta Barrionuevo González, M. Eugenia Peñas Lorite, José Manuel Gasalla Herraiz

**Affiliations:** Service of Clinical Biochemistry, Hospital Universitario Príncipe de Asturias, Madrid, Spain

**Keywords:** crystalluria, lupus, Malta cross, nephrotic syndrome, sulfadiazine

## Abstract

**Objectives:**

It is estimated that 29% of patients treated with sulfadiazine ultimately develop acute kidney failure. Diagnosis is based on urine sediment analysis.

**Case presentation:**

A 71-year-old woman with loss of visual acuity in the context of a flare of systemic erythematosus lupus (SEL). A diagnosis of acute retinal necrosis was established, pending etiological confirmation. Empirical treatment with sulfadiazine was initiated. Follow-up analyses included urine sediment, which revealed pH 6, 30–50 RBCs/field, urothelial cells and lower tract epithelial cells, hyaline casts, fatty casts or Maltese cross, and abundant sulfadiazine crystals. The finding was reported to the Unit of Nephrology, and treatment was immediately suspended.

**Conclusions:**

Sulfadiazine is an antibiotic of the family of sulfamides. Crystalization of sulfadiazine in the renal tubules may cause acute interstitial nephritis. These crystals adopt different shapes according to the metabolite that crystalizes: unaltered forms precipitate into dense, globular crystals, whereas in other cases, as in the case reported in this paper, crystals adopt a fan-shaped, shocks-of-wheat morphology.

## Case presentation

We report the case of a 71-year-old woman diagnosed with systemic erythematosus lupus (SEL) who presented at the Unit of Ophthalmology with a sudden loss of visual acuity in one eye. Upon diagnosis of acute retinal necrosis, the patient was hospitalized.

The patient had a history of retinal vasculitis with serous retinal detachment and grade-4 diffuse proliferative lupus nephritis, which was categorized as chronic kidney disease (CKD G3) on admission, based on the presence of creatinine levels of 1.4 mg/dL, urea 70 mg/dL, estimated glomerular filtrate (GF) (CKD-EPI) of 42 mL/min/1.73 m^2^, and proteinuria of 28 g/24 h within nephrotic range. Regular medication included prednisone 60 mg/24 h at decreasing doses, mycophenolate mofetil 3 g OD, and cycles of cyclophosphamide 900 mg every 3 weeks.

On admission due to diagnosis of retinal necrosis, and pending microbiological evaluation, empirical treatment with trimetoprim–sulfametoxazol (TMT-SMX) 160 mg/800 mg twice daily was initiated on suspicion of ocular toxoplasmosis. The appearance of skin lesions in the lower limbs raised suspicion of TMT-SMX-mediated leukocytoclastic vasculitis, which led to immediate treatment suspension and replacement with standard treatment for toxoplasmosis, pyrimethamine 50 mg OD and sulfadiazine 1 g four times daily [1, 2].

**Figure 1:**
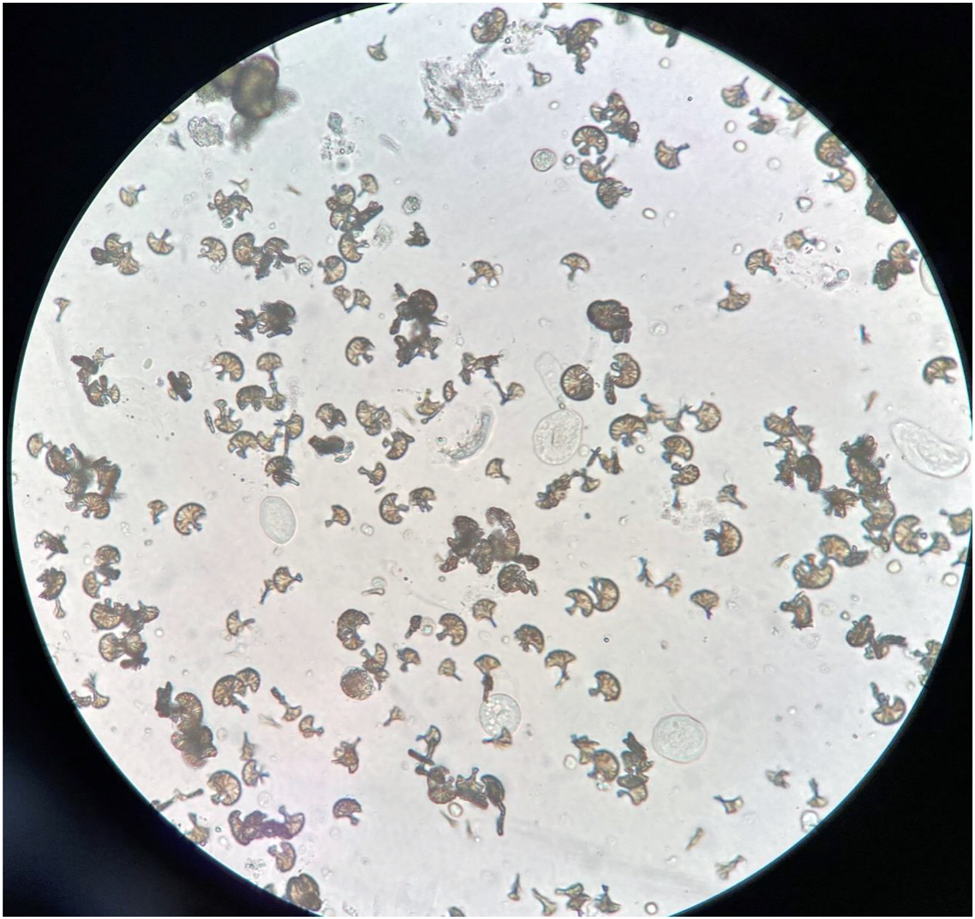
Sulfadiazine crystals in urine sediment, 40×. (Picture taken by authors).

**Figure 2:**
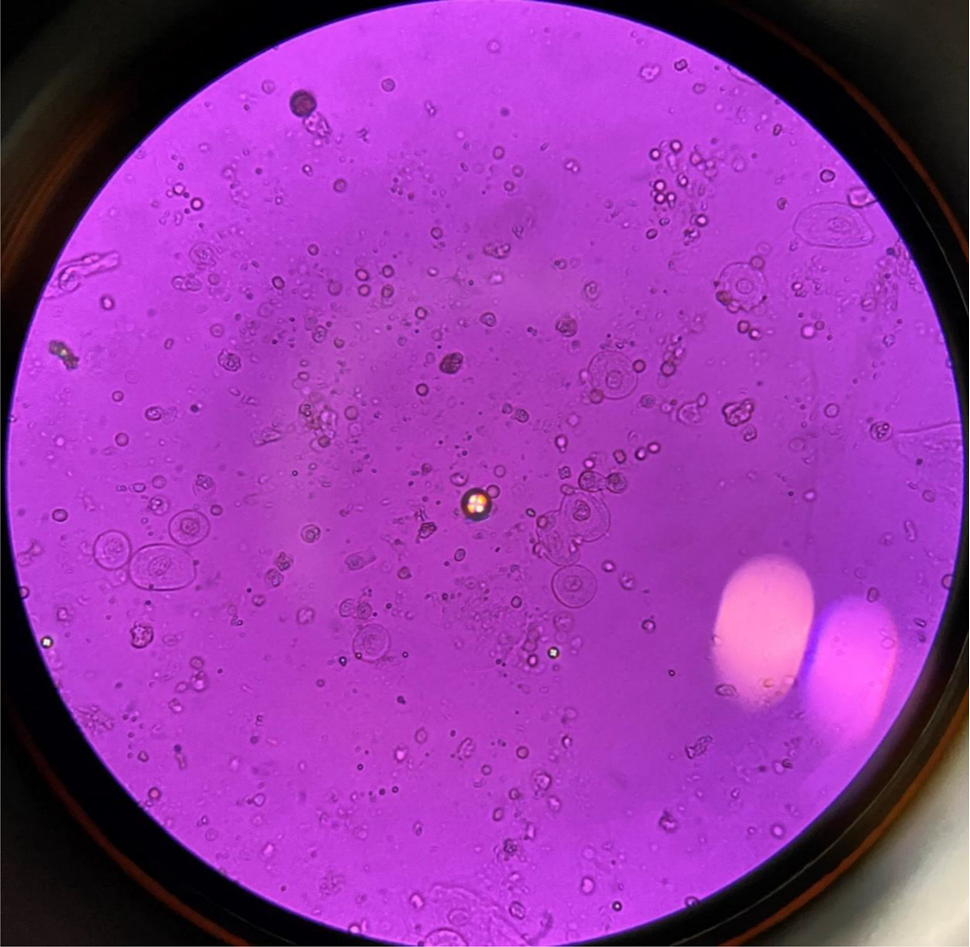
Malta cross bodies in the urine of a patient with nephrotic syndrome, 40×. (Picture taken by authors).

Following initiation of treatment, the patient experienced a sudden worsening of kidney function, with creatinine reaching 2.2 mg/dl and an estimated GF (CKD-EPI) of 21 mL/min/1.73 m^2^. Urine sediment revealed pH 6, 30–50 RBCs/field, urothelial cells and lower tract epithelial cells, hyaline casts, fatty casts or Maltese cross, and abundant sulfadiazine crystals and oval fat bodies (Figures 1, 2). In the light of these findings, the Unit of Nephrology was alerted.

## Discussion

Sulfadiazine crystals in the form of shocks of wheat with eccentric binding originate from crystalization of the metabolite acetyl sulfadiazine in the liver [3, 4]. Several factors favor precipitation of this drug, including acid urinary pH, hypoalbuminemia, conditions inducing volume depletion, drug concentration in urine, dose administered, and concomitant treatment with drugs that acidify urine [5, 6].

Treatment involves urine alkalization and fluid replacement to maintain daily diuresis above 2 L. The drug is withdrawn, and resuming therapy is considered when kidney function is restored to normal.

Oval fat bodies are drops of fat of different sizes, generally small, that originate from the rupture of fat-rich epithelial cells. When observed under a polarizing microscope, drops containing cholesterol have a characteristic Malt cross appearance. They are found in different tubulointerstitial conditions, being highly specific of nephrotic syndrome.

With regard to the case reported here, the complexity of the underlying condition of the patient made therapeutic management difficult. The precipitating factor was not urine pH, but a combination of factors related to her pre-existing disease, such as the use of diuretics, hypoalbuminemia, and volume depletion.

Sulfadiazine was immediately suspended and never resumed, due to the poor course of kidney disease. Microbiological analysis confirmed a herpetic etiology of retinal necrosis.

Withdrawal of diuretics and volume repletion was progressive, partly due to the poor course of kidney disease in the patient.
